# Synthesis and Properties of Silica and Alginate Hybrid Aerogel Particles with Embedded Carbon Nanotubes (CNTs) for Selective Sorption

**DOI:** 10.3390/ma12010052

**Published:** 2018-12-24

**Authors:** Natalia Menshutina, Pavel Tsygankov, Sviatoslav Ivanov

**Affiliations:** International Science and Educational Center for transfer of pharmaceutical and biotechnologies, Mendeleev University of Chemical Technology of Russia, Miusskaya pl. 9, 125047 Moscow, Russia; chemcom@muctr.ru (N.M.); s.ivanov@tbcorp.ru (S.I.)

**Keywords:** aerogel, aerogel composite, adsorption

## Abstract

The manuscript describes methods for producing hybrid silica microparticles and hybrid alginate beads with carbon nanotube (CNT) contents up to 4.5 and 30 wt.%, respectively. Silica hybrid aerogel microparticles with embedded nanotubes were obtained using a two-stage sol–gel method with a gelling process in an oil-emulsion. Alginate hybrid aerogels with embedded nanotubes were obtained using cross-linking reactions. The following methods were used to measure the structural characteristics of obtained materials: nitrogen adsorption porosimetry, scanning electron microscopy (SEM), and others. It is shown that specific surface area and pore volume increase with the increase of CNT content in silica aerogel microparticles. Obtained aerogels were tested as adsorbents for argon–oxygen separation. The alginate hybrid aerogel with 30 wt.% CNT content has the best argon adsorption selectivity.

## 1. Introduction

Aerogels are the lightest of the known solid materials and have a huge potential for use in various applications. However, aerogels have several disadvantages that limit their use. Depending on the aerogel type, the following properties have to be considered: low strength (silica aerogel), low electrical conductivity, and chemical instability to aggressive electrolytes. In order to improve certain aerogel characteristics, different excipients have to be added. Aerogels consisting of two or more different materials are the most promising [1], for example, aerogels with embedded carbon nanotubes (CNTs). The use of various raw materials allows the unique properties of aerogels to be maintained, such as: low density, high porosity, and specific surface area; and at the same time gives the material new functional properties, such as: strength, electrical conductivity, hydrophobicity, sorption, and selectivity. Sedova et al. [2] reported their investigation of the effects of tungsten disulfide nanotubes on the mechanical properties of silica aerogel. Several studies [3,4] described improvements of the mechanical properties of silica aerogels caused by addition of CNTs. In a study by Huang et al. [5], excellent adsorption capacities for various oils of a composite multiwall CNT–silica aerogel were shown.

During the production of hybrid aerogels with nanotubes, CNTs tend to form aggregates due to the action of van der Waals forces. The formation of aggregates affects the structure of hybrid aerogels and, as a result, their characteristics. Control of the aggregation process allows for the production of materials with the required properties. The distribution of nanotubes in a material is influenced by various factors, including the chemical composition of the surface, the dimensions, the synthesis method, and the concentration of nanotubes [6]. In addition, the type of polymer matrix and its physicochemical properties also affect the uniformity of distribution. A literature survey found that the following approaches and their combinations are used to obtain a uniform nanotube distribution: mechanical methods (calendering, use of ball mills, homogenization, and ultrasonic treatment), and the use of surface-active substances (surfactants). Functionalization of the CNT surface can also improve distribution. Piñero et al. [7] reported that they managed to obtain homogeneous silica–CNT hybrid aerogels using rapid gelation with precise control of the solution’s pH and the use of ultrasound.

In our previous paper, we described a production process for aerogel monoliths with embedded nanotubes and obtained the characteristics of the materials [8]. This paper is devoted to describing the methods for producing aerogel particles with embedded CNTs. The use of the obtained aerogel particles as sorbents for argon, nitrogen, and oxygen was also studied.

## 2. Materials and Methods

### 2.1. Materials

Tetraethoxysilane (TEOS, Fluka, Munich, Germany) was used as a silica precursor. Its purity was above 99%. Other materials included citric acid (purity 99.5%, Sigma-Aldrich, St. Louis, MO, USA), 2-propanol (purity 99.5%, Sigma-Aldrich), ammonium hydroxide solution (28.0–30.0% NH_3_ basis, Sigma-Aldrich), and the non-ionic surfactant Triton x-100 (Sigma-Aldrich). Sodium alginate was used as a precursor for alginate aerogel and was purchased from RusChem (Moscow, Russia). Anhydrous calcium chloride (RusChem) was used in aqueous solutions as a cross-linking agent. Carbon dioxide with a purity of >99% was used for supercritical drying.

Nanotubes with the following characteristics were used to prepare hybrid aerogels: chemical composition (C > 90 wt.%, O: 1–6 wt.%, Cl < 1 wt.%, Co < 5 wt.%, Mo < 1 wt.%), bulk density 0.15 g/cm^3^, diameter 30–40 nm, length 1 μm, and Brunauer–Emmett–Teller (BET) specific surface area 1200–1500 m^2^/g. Multi-walled carbon nanotubes (MWCNTs) were purchased from Global & Co, Moscow, Russia.

### 2.2. Synthesis of Silica–CNT Hybrid Aerogel Microparticles

**Figure 1:**
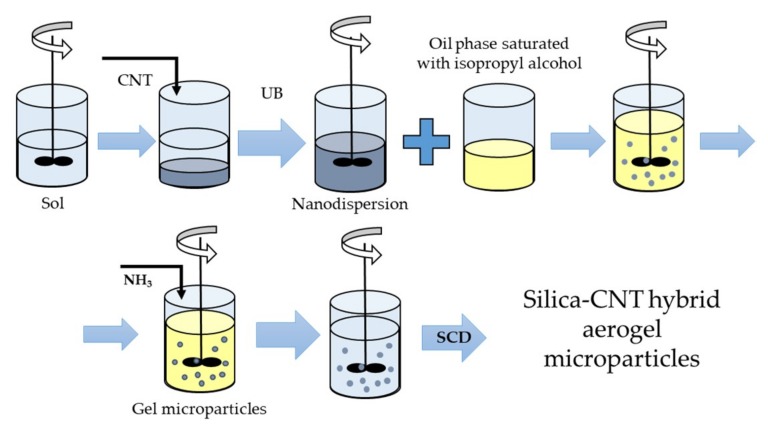
Synthesis of silica–carbon nanotube (CNT) hybrid aerogel microparticles. UB is ultrasonic bath; SCD is supercritical drying.

The general scheme of the method used for producing spherical silica aerogel microparticles with embedded carbon nanotubes is shown in Figure 1. TEOS was used as a silica component source. Citric acid, isopropyl alcohol, and ammonia solution were utilized, respectively, as an acid catalyst, solvent, and basic catalyst.

At the sol-forming stage, TEOS was mixed with isopropyl alcohol, and 0.1 M aqueous citric acid solution was added. The resulting solution was stirred for 10–15 min with a magnetic stirrer and left for 24 h at room temperature. A surfactant was then added to the resulting sol and the resulting mixture was stirred for 20 min. Then CNTs were added while stirring in an ultrasonic bath (UB) for 2 h. The concentration of CNTs was varied from 0 to 4.5 wt.% during the research. The subsequent increase in the CNT concentration at the emulsification stage, carried out in an oil, caused the sedimentation of CNTs, and gelation occurred in a non-uniform way.

The emulsification of the sol was conducted in an oil phase saturated with isopropyl alcohol while stirring at a constant speed (1000 rpm) for 60 min. The mean particle diameter was 100 µm at 1000 rpm. The emulsion formation was a mandatory stage for spherical gel microparticle production. This stage allowed a large number of stable spherical microparticles to be obtained.

For the gelation stage, 0.5 M ammonia solution was added dropwise with constant stirring. As a result, particles of the dispersed phase were formed. The gelation of the dispersed phase finished in 20–30 min.

The obtained gel particles were placed in isopropyl alcohol for one day to remove unreacted substances. After this procedure, spherical gel microparticles were ready for supercritical drying (SCD), which was the final stage of the production process.

The final stage of the production of silica–CNT hybrid aerogel microparticles was supercritical drying in carbon dioxide media.

### 2.3. Synthesis of Alginate–CNT Hybrid Aerogel Beads

**Figure 2:**
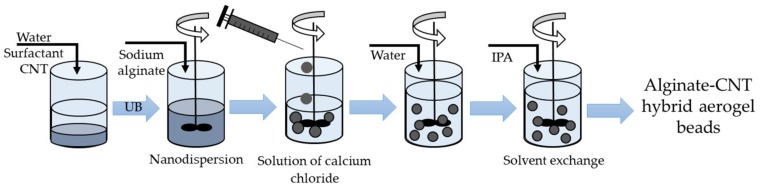
Synthesis of alginate–CNT hybrid aerogel beads. IPA is isopropyl alcohol.

For the production of hybrid alginate-based aerogel beads with embedded CNTs, the method shown in Figure 2 was used.

The surfactant was added to the water and stirred for 20 min. Then CNTs were added, and the ultrasonic treatment was conducted for 2 h. The concentration of CNTs was varied from 0 to 30 wt.% during the study. Sodium alginate was added to the obtained nanodispersion and stirred for 24 h.

The resulting solution of sodium alginate was added dropwise, through a needle, to a solution of a crosslinking agent (calcium chloride), which was continuously stirred. Upon contact of the solutions, particle formation started. Alginate gelation occurred when alginate polymer chains cross-linked through the bonding of divalent cations Ca^2+^. Gel particles were then stored in a solution of calcium chloride for 24 h in order to complete the chemical reaction.

The obtained gel particles were washed with distilled water to remove excess calcium chloride. Further step-by-step solvent exchange with isopropyl alcohol (IPA) was carried out. Particles were put in a mixture “water–IPA” and aged for 4 h. At each next step, the alcohol concentration increased from 30 to 60, 90, and 100 wt.%. Exchange with 100 wt.% of IPA was carried out twice.

As was the case with silica–CNT hybrid aerogels, the final step of production of alginate-based aerogel microparticles with embedded CNTs was supercritical drying.

More detailed information about aerogel bead synthesis is provided in our previous paper [8].

### 2.4. Supercritical Drying

**Figure 3:**
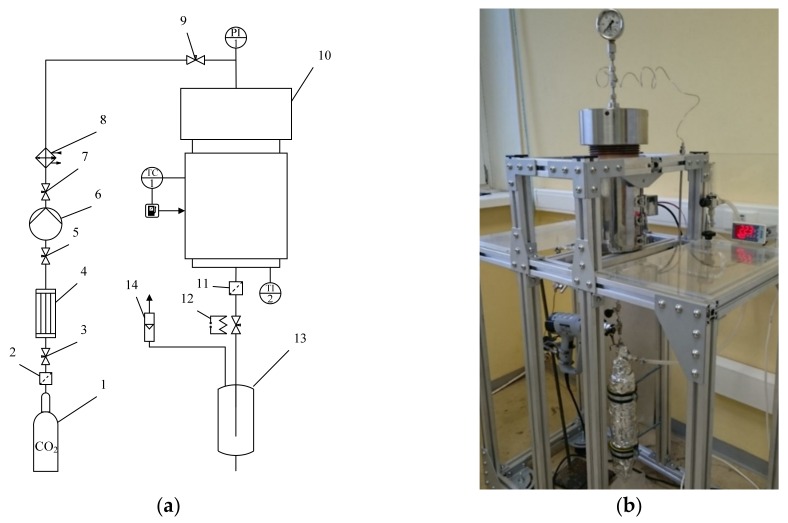
Supercritical drying experimental setup: (**a**) flowsheet of supercritical drying experimental setup: 1—vessel with liquid СO_2_ (60 bar); 2, 11—micro filters; 3, 5, 7, 9—needle valves; 4—condenser; 6—pneumatic high-pressure pump; 8—thermostat; 10—2-L high-pressure autoclave; 12—decompression valve with heating; 13—separator; 14—flowmeter; (**b**) appearance of supercritical drying experimental setup.

A flowsheet of the supercritical drying experimental setup is shown in Figure 3.

The liquid CO_2_ was supplied to the system from a vessel (1) at ambient temperature and a pressure of 60 atm. In order to ensure that the carbon dioxide was supplied to a pump (6) in the liquid state, the carbon dioxide was cooled in a condenser (4) below 5 °C. To build up pressure, a pneumatic high-pressure pump (6) manufactured by Maximator (Nordhausen, Germany) was used. The preheating of carbon dioxide was carried out in a thermostat (8). Then carbon dioxide was fed to a 2-L high-pressure autoclave (10). The flow of carbon dioxide was controlled using a decompression valve with heating (12), which is necessary to prevent the valve from freezing. A separator (13) was used to separate the liquid phase. To determine the volumetric flow rate of carbon dioxide after separation of the liquid phase, a flowmeter (14) was used. The temperature inside the autoclave was controlled by a temperature controller (TC). The temperature inside the autoclave was determined using a KTL-01 (type K) thermocouple (accuracy of ±0.1 °C), and the pressure was measured using a Wika manometer (accuracy of ±1 bar).

Samples were loaded into the autoclave. The autoclave was hermetically sealed and liquid CO_2_ was fed into it. The drying process was as follows: solvent displacement (IPA) from the reactor at 120 bar, temperature 25 °C, and rate 40 NL/min; supercritical drying within 6 h at pressure 120–140 bar, temperature 45 °C, and rate 20 to 25 NL/min; pressure release within 1 h at the rate 40 NL/min.

### 2.5. Characterization

The nitrogen adsorption–desorption isotherms were measured at −196 °C using a volumetric apparatus (ASAP 2020, Micromeritics, Norcross, GA, USA). The specific surface area was calculated using the BET method for isotherm linear range, and the total sorption mesopore volume was obtained at P/P0 = 0.95. Pore diameters were determined using the Barrett–Joyner–Halenda (BJH) algorithm. The BJH algorithm uses a modified Kelvin equation to link the removed adsorbed material from pores with the pore sizes. Scanning electron microscopy imaging was performed on a SEM (Helios NanoLab 600I with a Pt-coated sample, Thermo Fisher).

**Figure 4:**
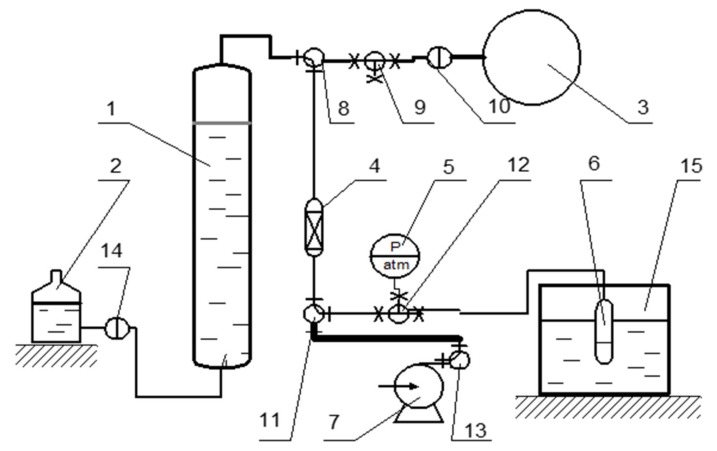
Volumetric analytical setup: 1—calibrated nitrometer, 2—liquid compensator, 3—gas chamber, 4—dehumidifier, 5—vacuometer, 6—glass closed chamber, 7—vacuum pump, 8–14—valves, 15—thermostat.

The equilibrium quantities of adsorbed nitrogen, argon, and oxygen were measured using the volumetric method at 298 K and atmospheric pressure. A flowsheet of the volumetric analytical setup is shown in Figure 4.

A sample was placed in a glass closed chamber (6) connected via a valve (12) to a setup consisting of a calibrated nitrometer (1), a liquid compensator (2), and a gas chamber (3). The system was evacuated with a vacuum pump (7), and then the nitrometer was filled with a gas. The chamber (6) with the sample was evacuated for 30 min, and then the gas was supplied via the valve (12) to the chamber from the nitrometer. The pressure in the system was kept at atmospheric level by equalizing the liquid in the nitrometer (1) and the liquid compensator (2). The amount of absorbed gas was measured in time by observing liquid level changes in the nitrometer (1). The equilibrium capacitance was calculated using the experimental data. Each experiment was repeated thrice to obtain converged results.

## 3. Results and Discussion

### 3.1. Characteristics of Silica–CNT Hybrid Aerogel Microparticles

**Figure 5:**
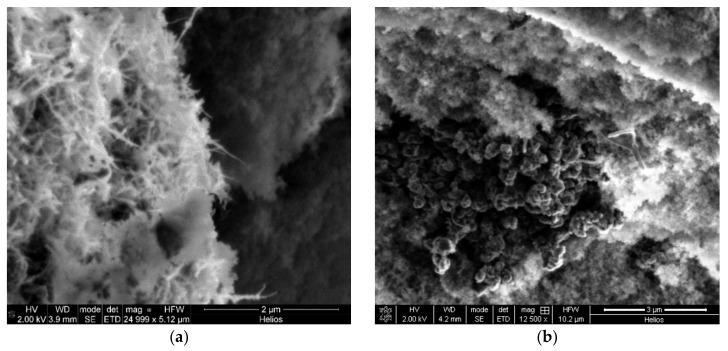
SEM image of silica–CNT hybrid aerogel: (**a**) microparticles with a CNT weight percentage of 4.5 wt.%; (**b**) aggregation of CNTs.

Silica–CNT hybrid aerogel microparticles were obtained. Silica-based aerogel particles have diameters of about 100 µm. These particles were produced using an oil-emulsion method. The following concentrations of nanotubes were used: 0, 0.2, 1.0, and 4.5 wt.%. A scanning electron microscope image of the silica–CNT hybrid aerogel microparticles is shown in Figure 5.

As seen in Figure 5a, there are a lot of carbon nanotubes directly on the surface of the particle (fiber-like structures of bright color are visible). This explains the increase in the specific surface area. At the same time, Figure 5b shows agglomerates of CNTs (dark structures in the left side of the image), which form when the CNT concentration is high.

**Figure 6:**
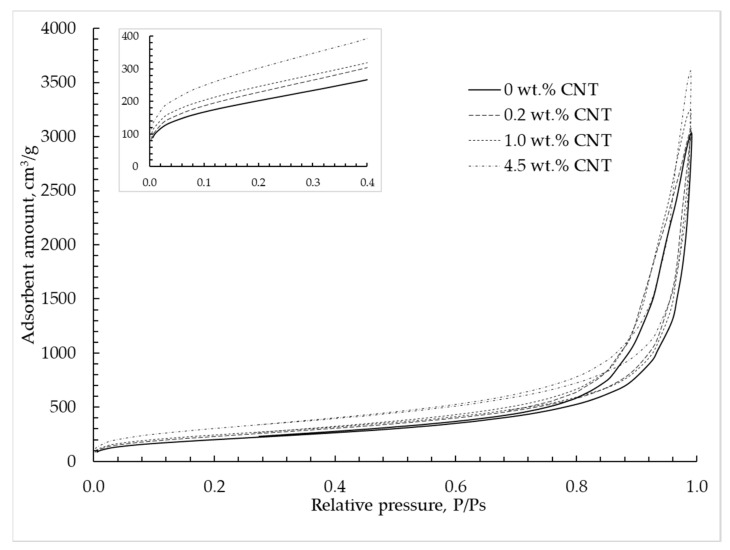
Adsorption–desorption isotherms are provided at 77 K in silica hybrid aerogel microparticles with embedded CNTs.

The nitrogen adsorption–desorption isotherm at 77 K for silica–CNT hybrid aerogel microparticles is shown in Figure 6 and corresponds to type IV according to the IUPAC classification [9]. This type of isotherm is typical for mesoporous materials in which capillary condensation is observed. With a CNT mass concentration increase, an increase of nitrogen adsorption is observed, which indicates an increase of the total pore volume.

**Figure 7:**
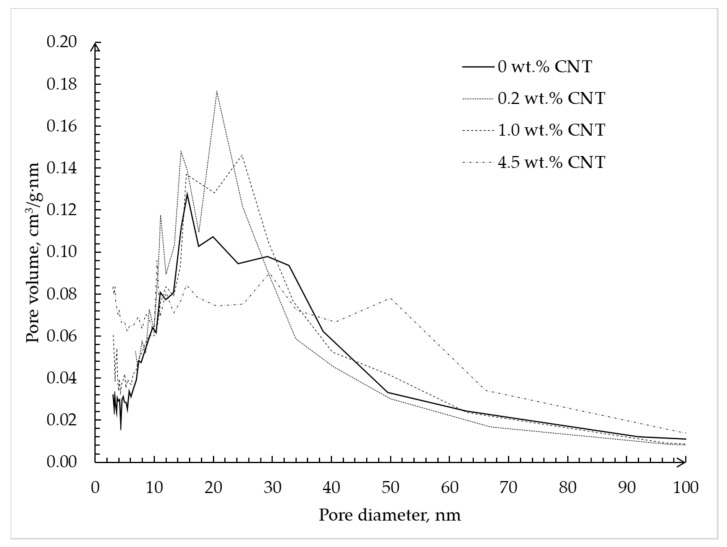
Pore size distribution calculated using the Barrett–Joyner–Halenda (BJH) method based on desorption isotherms of silica hybrid aerogel microparticles with embedded CNTs.

Figure 7 shows the pore size distribution of silica–CNT hybrid aerogel microparticles obtained using the BJH method.

The properties of the produced silica–CNT hybrid aerogel microparticles are provided in Table 1 (specific surface area (S_sp_), specific pore volume (V_t_), specific mesopore volume (V_m_)).

The data in the table show that an increased concentration of nanotubes in silica–CNT hybrid aerogel microparticles leads to an increase of the specific surface area, the specific pore volume, and the specific mesopore volume. This is due to the peculiarity of structure formation (gelation is conducted with constant stirring within the dispersed phase). Thus, the formed microparticles have a large number of open CNTs on their surface (fiber-like structures of bright color in Figure 5a), which leads to an increase in the values of the properties.

### 3.2. Characteristics of Alginate–CNT Hybrid Aerogel Beads

**Figure 8:**
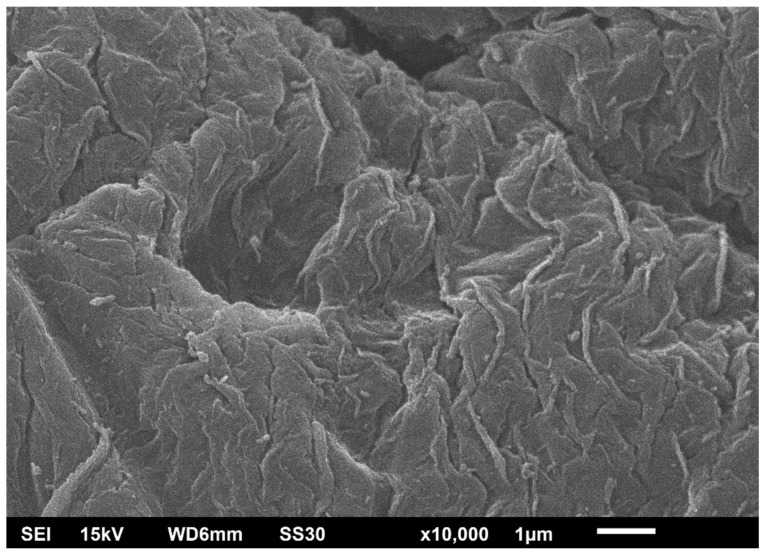
SEM image of alginate–CNT hybrid aerogel microparticles with a CNT concentration of 7.5 wt.%.

Alginate–CNT hybrid aerogel beads were obtained. Alginate-based particles have diameters of about 2–3 mm. They were produced via dropping. The following concentrations of nanotubes were used: 0, 7.5, and 30 wt.%. A scanning electron microscope image of alginate–CNT hybrid aerogel microparticles is shown in Figure 8.

In Figure 8, the surface of an alginate particle is shown. It can be seen that no CNTs are visible. It seems that the whole surface is composed solely of alginate, and all CNTs are embedded in the structure of the aerogel.

**Figure 9:**
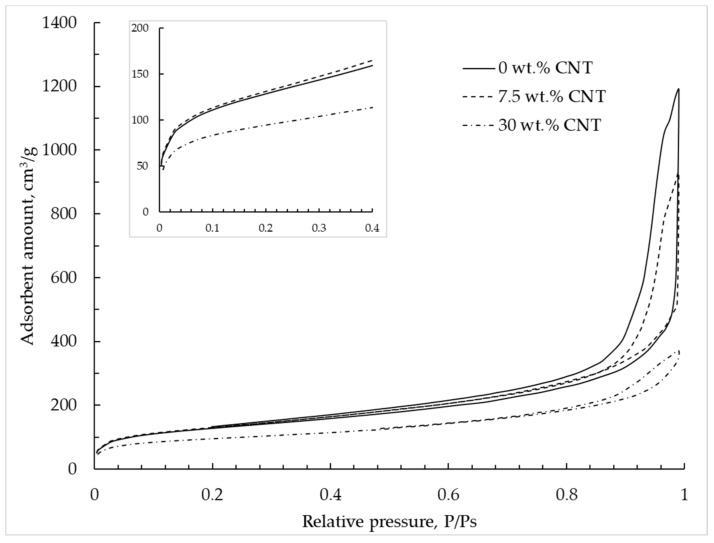
Adsorption–desorption isotherms are provided at 77 K in alginate hybrid aerogel beads with embedded CNTs.

The nitrogen adsorption–desorption isotherm at 77 K for alginate–CNT hybrid aerogel beads is shown in Figure 9 and corresponds to type IV according to the IUPAC classification [9]. With an increase of CNT mass concentration, a decrease of adsorbed nitrogen was observed, which indicates a decrease of the total pore volume. With an increase of CNT concentration, a decrease of the hysteresis loop region was observed, which indicates a decrease of the mesopore volume.

**Figure 10:**
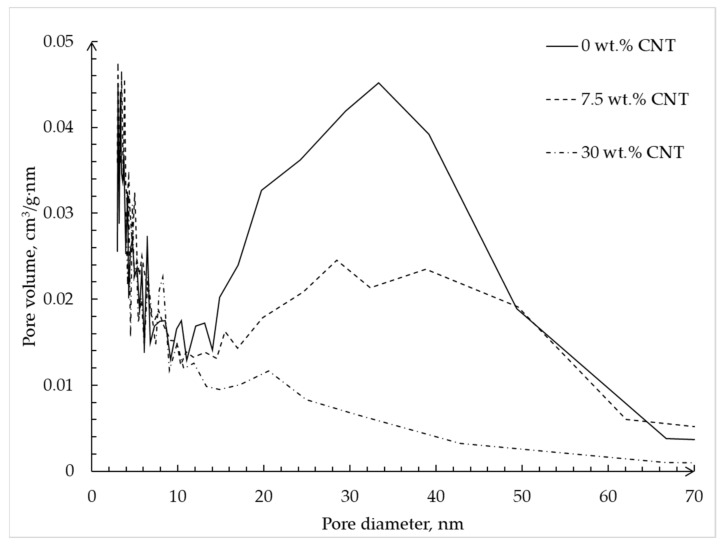
Pore size distributions calculated using BJH method based on desorption isotherms of alginate hybrid aerogel beads with embedded CNTs.

Figure 10 shows the pore size distribution of alginate–CNT hybrid aerogel beads obtained using the BJH method.

The plot shows that with an increase in CNTs’ mass fraction in the alginate–CNT hybrid aerogel beads, a decrease of the total volume of pores with a diameter of 35–40 nm, which corresponds to the diameter of the carbon nanotube, is observed, but there is no visible change over the interval of 0–15 nm. This fact confirms the hypothesis that CNTs are embedded in free pores with a size of 30–40 nm, which corresponds to the diameter of the tubes. In addition, we can conclude that when adding 30 wt.% of CNTs, the complete filling of pores with a diameter of 30–40 nm occurs.

Properties of obtained alginate–CNT hybrid aerogel beads are shown in Table 2 (specific surface area (S_sp_), specific mesopores volume (V_m_)) in comparison with the sample without CNTs.

The addition of CNTs up to 7.5 wt.% did not significantly affect the specific surface area. As mentioned above, the nanotubes were embedded in mesopores with a size of 30–40 nm. With an increase in the CNT concentration up to 30 wt.%, a decrease in the specific surface area and volume of mesopores was observed due to the aggregation of the CNTs and the influence on the aerogel’s microstructure.

### 3.3. The Study of the Equilibrium Adsorption Capacities of Hybrid Aerogels with Embedded CNTs with Respect to Nitrogen, Oxygen, and Argon

The sample vessel was evacuated after each measurement. The relative error of the applied method did not exceed 5%. The gas purity was as follows (vol.%): nitrogen (superior grade) 99.9, oxygen (superior grade) 99.9, and argon (superior grade) 99.9. The separation factor was calculated as the ratio of the argon to oxygen equilibrium capacity.

Table 3 shows the average values of the equilibrium quantities of the air macrocomponents adsorbed by the samples of silica-nanotube and alginate-nanotube hybrid aerogels (based on the results of two measurements) and the calculated values of the argon–oxygen mixture separation factor.

All studied samples were characterized by low equilibrium nitrogen adsorption, and the addition of carbon nanotubes into the aerogels’ structure led to a decrease of the equilibrium capacity.

The equilibrium quantities of adsorbed oxygen and argon for the pure alginate aerogel were quite close, but this sample also showed some selectivity with respect to the argo. Sample No. 2, with a CNT concentration of 7.5 wt.%, had a higher equilibrium quantity of adsorbed oxygen than of argon, and sample no. 3, containing 30 wt.% of CNTs, showed twice as much capacity with respect to argon than to oxygen: the mixture separation factor was 2.0. The separation factor decreased to 1.6 after dehydration in a stream of nitrogen at a temperature of 50 °C.

Based on Table 3, it can be concluded that silica–CNT hybrid aerogels exhibit a slight selectivity to argon. The highest value of the separation factor for an argon–oxygen mixture, equal to 1.3, was observed for sample no. 6 with a CNT content of 1.0 wt.%.

As the obtained results show, the addition of carbon nanotubes into aerogel structures led to an increase of argon adsorption by a factor of 1.2–1.3 for silica aerogels and by a factor of 2 for alginate aerogels. This is probably a consequence of the partial blocking of the active oxygen sorption centers by carbon nanotubes.

## 4. Conclusions

This study determined the changing patterns of the properties of hybrid aerogels with embedded CNTs. An increase in the nanotube concentration leads to an increase in the specific surface area and the pore volume of silica–CNT hybrid aerogels produced using the oil-emulsion method, which relates to the structure formation features. The produced microparticles have a large number of “open” CNTs on their surface. It was shown that with an increase of the CNTs’ concentration above 4.5 wt.%, the gelation process was not uniform. Alginate–CNT hybrid aerogels were obtained using the dropping method. With an increase of the CNT concentration, a decrease in the volume of pores with a diameter of 35–40 nm—which corresponds to the diameter of the CNTs—was observed. It was shown that hybrid aerogels with embedded carbon nanotubes have selective sorption of argon. An addition of 30 wt.% of CNTs to the alginate aerogel leads to a two-fold increase of argon adsorption compared to oxygen. This is probably a consequence of the partial blocking of the active oxygen sorption centers by carbon nanotubes.

## Figures and Tables

**Table 1:** Properties of silica–CNT hybrid aerogel microparticles (S_sp_ is specific surface area, V_t_ is specific pore volume, and V_m_ is specific mesopore volume).

CNT (wt.%)	S_sp_ (m^2^/g)	V_t_ (cm^3^/g)	V_m_ (cm^3^/g)
0	737	4.67	0.30
0.2	849	4.75	0.33
1.0	886	5.01	0.38
4.5	1097	5.56	0.43

**Table 2:** Properties of alginate hybrid aerogel beads with CNT.

CNT (wt.%)	S_sp_ (m^2^/g)	V_m_ (cm^3^/g)
0	459	1.84
7.5	449	1.44
30	317	0.58

**Table 3:** Equilibrium quantities of adsorbed nitrogen, oxygen, and argon for hybrid aerogels with embedded CNTs.

No.	Sample	Equilibrium Adsorption at 25 °C and 0.1 MPa, cm^3^/g			Ar–O_2_ Separation Factor
		N_2_	Ar	O_2_	
1	Alginate aerogel	6.6	20.9	19.9	1.1
2	Alginate aerogel + 7.5 wt.%	6.5	20.4	25.9	0.8
3	Alginate aerogel + 30 wt.%	4.5	18.5	9.3	2.0
4	Silica aerogel	2.4	16.0	15.7	1.0
5	Silica aerogel + 0.2 wt.%	1.7	9.8	8.3	1.2
6	Silica aerogel + 1.0 wt.%	1.1	8.0	6.3	1.3
7	Silica aerogel + 4.5 wt.%	1.2	7.6	6.7	1.1

## References

[1] Smirnova I., Gurikov P. (2018). Aerogel production: Current status, research directions, and future opportunities. J. Supercrit. Fluids.

[2] Sedova A., Bar G., Goldbart O., Ron R., Achrai B., Kaplan-Ashiri I., Brumfeld V., Zak A., Gvishi R., Wagner H. (2015). Reinforcing silica aerogels with tungsten disulfide nanotubes. J. Supercrit. Fluids.

[3] Hamilton C.E., Chavez M.E., Duque J.G., Gupta G., Doorn S., Dattelbaum A.M., Obrey K.A.D. (2010). Carbon nanomaterials in silica aerogel matrices. MRS Online Proc. Libr. Arch..

[4] Zhang Y., Shen Y., Han D., Wang Z., Song J., Niu L. (2006). Reinforcement of silica with single-walled carbon nanotubes through covalent functionalization. J. Mater. Chem..

[5] Huang J., Liu H., Chen S., Ding C. (2016). Hierarchical porous MWCNTs-silica aerogel synthesis for high-efficiency oily water treatment. J. Environ. Chem. Eng..

[6] Atif R., Inam F. (2016). Reasons and remedies for the agglomeration of multilayered graphene and carbon nanotubes in polymers. Beilstein J. Nanotechnol..

[7] Piñero M., del Mar Mesa-Díaz M., de los Santos D., Reyes-Peces M.V., Díaz-Fraile J.A., de la Rosa-Fox N., Esquivias L., Morales-Florez V. (2018). Reinforced silica-carbon nanotube monolithic aerogels synthesised by rapid controlled gelation. J. Sol-Gel Sci. Technol..

[8] Menshutina N., Ivanov S., Tsygankov P., Khudeev I. (2017). Synthesis and characterization of composite materials “aerogel-MWCNT”. J. Sol-Gel Sci. Technol..

[9] Thommes M., Kaneko K., Neimark A.V., Olivier J.P., Rodriguez-Reinoso F., Rouquerol J., Sing K.S. (2015). Physisorption of gases, with special reference to the evaluation of surface area and pore size distribution (IUPAC Technical Report). Pure Appl. Chem..

